# Qualitative findings from an exploratory trial of the Healthy Lifestyles Programme (HeLP) and their implications for the process evaluation in the definitive trial

**DOI:** 10.1186/1471-2458-14-578

**Published:** 2014-06-09

**Authors:** Jenny J Lloyd, Katrina M Wyatt

**Affiliations:** 1 Institute for Health Research, University of Exeter Medical School, University of Exeter, Exeter, UK

**Keywords:** Feasibility, Acceptability, Exploratory trial, Process evaluation, Engagement, Behaviour change, Supportive contexts

## Abstract

**Background:**

Approximately one third of 10-11 year olds in England are now overweight or obese suggesting that population approaches are urgently required. However, despite the increasing number of school-based interventions to prevent obesity, results continue to be inconsistent and it is still unclear what the necessary conditions are that lead to the sustained behaviour change required to affect weight status. The Healthy Lifestyles Programme is a theoretically informed four phase multi-component intervention which seeks to create supportive school and home environments for healthy behaviours.

**Methods:**

A process evaluation has run alongside the exploratory trial of the Healthy Lifestyles Programme to ascertain the feasibility and acceptability of; the trial design (including the trial outcomes) and the HeLP Programme and whether it is able to engage schools, children and their families. Data was collected using interviews with teachers (n = 12) and parents (n = 17) and six focus groups with children (n = 47) and a questionnaire for parents of children in the intervention schools. Interview and focus group data relating to the intervention was analysed using framework analysis.

**Results:**

Four schools and 201 children participated in the exploratory trial. The data showed that the trial design was feasible and acceptable for schools and children. Three themes emerged for the data in relation to the acceptability and feasibility of the HeLP Programme (*value, compatibility with the curriculum and enjoyment*) and two themes emerged in relation to engagement *(‘knowledge and awareness’ and ‘taking messages on board’*). The latter could be broken down into 4 subthemes (‘*initiating discussion with family and friends’, ‘acceptance of family rules’, ‘increased responsibility’ and ‘the importance of the mode and agent of delivery’*). The use of highly inclusive and interactive delivery methods where the children were encouraged to identify with and take ownership of the healthy lifestyle messages were identified as important factors in motivating the children to take the messages home, seek parental support and initiate family lifestyle behaviour change.

**Conclusion:**

The process evaluation of the exploratory trial has not only provided evidence of the feasibility and acceptability of the Programme, it has also allowed an understanding of *how* HeLP engages schools, children and their families. These findings have informed the process evaluation for the definitive trial.

## Background

### Introduction

Childhood obesity is one of the most serious public health challenges of the 21^st^ century [1]. Data from England’s National Childhood Measurement Programme (NCMP) for 2011-2012 showed that 22.6% of children entering primary school were overweight or obese, rising to 33.9% when they leave primary school [2]. Recent UK trends suggest that the rate of increase in childhood obesity may have slowed [3], however the prevalence rates are still high and significantly higher than before 1980 [4]. Inequalities among children continue to increase, with the highest prevalence of overweight and obesity among those from poorer backgrounds [3]. Until recently, evidence on the incidence of overweight and obesity by age group has been limited, resulting in a lack of explicit guidance in choice of target population for obesity prevention [5]. However, data from a large prospective cohort study in the South West of England [6] showed that four year incidence of obesity was higher between the ages of seven and 11 years than between 11 and 15 years, suggesting that mid childhood may be an appropriate age in which to deliver prevention programmes.

As parents, families, the home and the school environment are important influences on children’s eating and physical activity behaviours [7], schools are viewed as a key setting in which to deliver prevention programmes. Not only do they have the organisational, social and communication structures in place to promote health [8] but they also have the potential to reach children and their families across the social spectrum. Schools can create supportive environments for healthy eating and regular physical activity [8] and teachers are generally supportive of such approaches [9]. Consequently, health researchers and guidelines all highlight the role of schools in obesity prevention [10,11].

The WHO’s Health Promoting School (HPS) framework advocates a holistic, settings-based approach, consisting of a cycle of steps to guide and implement change in a flexible manner with a focus on action in three areas; the curriculum; the school ethos/environment and links with families/communities [12]. This approach promotes the building of positive relationships with teachers, pupils and families in order to develop a sense of ‘school connection’ (a pupil’s sense of feeling part of his/her school, feeling valued and being treated fairly) [13,14] which has been linked to improvement in child health and wellbeing [15]. The importance of the quality of the social relationships and the school as a social institution in promoting child wellbeing has long been advocated by Rutter [16] and subsequent research exploring the impact of more specific school and classroom characteristics which influence pupil engagement in learning and social development has shown that student progress is positively associated with good relationships between teachers and pupils, opportunities for pupil participation and responsibility and support structures for teachers [17-20].

Despite this strong rationale for building trusting and supportive relationships at the level of the school, child and family in promoting child health and wellbeing, there has been a paucity of obesity prevention interventions that aim to positively affect relations within the school and within the family as well as trying to affect individual level processes such as improve knowledge and skills and modify norms [21].

A recent review by Khambalia and colleagues [22] examined the quality of evidence and findings from existing systematic reviews and meta-analyses of school-based obesity prevention programmes. All of the reviews recognised the heterogeneity of the studies including, participants, intervention and outcomes. Most interventions only reported the specifics of intervention components, with very little, if any, reporting on the school context and how this might interact with them. The only conclusions that could be drawn from this review were that interventions associated with a significant reduction of weight in children were of long term duration, focused on both diet and physical activity and included a family component. Unfortunately, no guidelines could be provided in relation to specific programme characteristics predictive of success.

A recent paper by Wells et al. [23] suggest that sufficient detail about context needs to be understood and reported in RCTs of complex interventions, in order for their transferability to be assessed. According to Plsek and Greenhalgh [24], context is continually evolving as it involves the interconnected actions of individuals, suggesting we need to develop adaptive interventions which can respond to their context. This also suggests that such interventions (and their components) should be characterised by their ‘function’ as well as their ‘form’ which are important considerations when assessing fidelity and manualising interventions [25].

From the outset, we sought to develop an intervention that could be adapted to the local school context. In line with the HPS framework we took a whole school approach and aimed to develop activities that impacted the school environment as well as specific behaviours of children and their families. First and foremost we sought to build supportive and trusting relationships by employing deliverers with specific skills and competencies, using the initial phase of the intervention to create a receptive context and by using engaging delivery methods to try and increase the uptake of the programme. It was agreed that each phase of the intervention should be defined in terms of its ‘function’ as well as its ‘form’ and that components within each phase could be adapted slightly to better fit the context in which it was being delivered. We believed that this approach would improve feasibility and acceptability and thus engagement with HeLP.

### The Development of HeLP

HeLP was developed using Intervention Mapping (IM) [26] which combines multiple theoretical and experiential perspectives with new research to assess and/or develop a potential set of possible solutions for a particular problem rather than defining a practice or research agenda around a single specific theory [27]. HeLP was developed to affect both the upstream and downstream influences on health behaviours as well as build relations to affect family behaviours [21].

A substantial amount of time in the first step of IM (needs assessment) was spent engaging teachers and the local education and health authority to understand the primary school system and how best to work with schools to deliver a programme of activities which children and their families would want to participate in. In order to define possible behaviour change techniques to employ within the intervention, we used the Information, Motivation and Behavioural Skills Model (IMB) as a guide [28]. This model was chosen as it linked closely to the selected determinants of our three key behavioural objectives; reducing sweetened fizzy drink consumption; increasing the proportion of healthy to unhealthy snacks consumed and reducing sedentary behaviours. In order to promote children’s access and availability of opportunity (a key determinant not considered in the IMB model), we sought to engage parents and offer them strategies through which they could directly (through parenting) or indirectly (through the creation of supportive environments) foster the development of healthy eating and activity behaviours among their children/family. In order to guide the sequential order in which the behaviour change techniques were to be delivered, we used the Health Action Process Model (HAPA) as a guide [29]. This phased model implies a clear order of distinct actions starting with establishing motivation, moving on to taking action followed by maintaining motivation.

Early development of HeLP involved two pilot studies involving 200 children. The aim of pilot one was to trial methods in which to deliver the selected behaviour change techniques to promote the healthy lifestyle messages to 8-11 year olds in one primary school and the aim of pilot two was to adapt the intervention based on pupil, teacher and parent responses from pilot one and carry out a before and after study in another primary school. Most school-based obesity prevention interventions to date have used traditional delivery methods such as education lessons to teach children about the importance of healthy nutrition and physical activity as opposed to methods where the child actively engages with the messages [22]. One exception is an intervention that used theatre as a novel delivery approach [30]. This intervention, aimed at low income children and their parents, showed that an after school theatre programme motivated and engaged both parents and children and increased awareness of the need for making changes, however, on its own, was not sufficient to change behaviours. The authors concluded that further development should be made to incorporate this novel delivery method into more comprehensive programmes with both educational and environmental components. In developing HeLP we were mindful that the children themselves, if suffciently motivated, were a key resource in taking messages home to their families, encouraging their parents to attend activities and in affecting change at home.The main delivery method trialled in pilot one, therefore, was interactive drama as it showed promise in promoting positive attitudes towards a number of health behaviours [31] and was a means of delivering the range of behaviour change techniques selected during the IM process.

The drama was built around a framework of four characters with whom the children could identify and designed to encourage the children to co-create scenes and come up with ideas to help the characters lead a more healthy lifestyle. Key findings to emerge from the data in the first pilot were that the year 5s (9-10 year olds) were most receptive to the messages and engaged their parents to the greatest extent and that it was more feasible for the school to run the HeLP activities in year 5 rather than year 6. As a result, year 5 was selected as the target group. The children were unanimous in their enjoyment of the drama activities, with many parents reporting that their child had talked about the activities at home, encouraged other family members to make changes and wanted their parents to come into the school to view programme activities. The IM process and the early piloting outlined above are published in detail elsewhere [26,32].

### The HeLP intervention

Following the early pilots, the intervention was adapted and refined to produce a programme consisting of four phases each with multiple components and a range of delivery methods. The intervention targets the year 5 children (although some components are also delivered to the whole school) and runs over three school terms (spring and summer term of year 5 and autumn term of year 6). The aim is to deliver a general healthy lifestyle message encouraging a healthy energy balance with a focus on changing three specific behaviours relating to energy intake and expenditure; decreasing the consumption of sweetened fizzy drinks; increasing the ratio of healthy to unhealthy snacks consumed, and reducing screen-based activities*.* We adopted the ‘80/20’ mnemonic, which suggests we should be active and eat healthily 80% of the time. This ‘tag’ came out of the early pilots as parents and children found that it acted as a trigger for remembering the three key behaviours. Phase 1, *Creating a Supportive Context* aims to establish relationships, and raise awareness of HeLP, setting the foundation for the successful delivery of subsequent components. Phase 2 is the intensive *Healthy Lifestyles Week* involving education lessons (delivered by the class teacher) and interactive drama activities (delivered by a local drama group). The drama framework includes four characters (Disorgaised Duncan, Fooball Freddie, Snacky Sam and Active Amy), each represented by one of the actors, whose attributes related to the three key behaviours. Children choose which of the characters they most resemble then work with that actor to help the character learn to change their behaviour. Phase 3 is *Personal Goal Setting with Parental Support* and encourages the children to focus on themselves by setting goals based on the HeLP messages with their parents. Phase 4 is *Reinforcement Activities* and involves a range of components to refocus the children and their parents on the HeLP messages and behaviour change.

Table 1 shows each phase of HeLP, its function, the BCTs used and the component (form) and agent of delivery. Each phase of HeLP has been designed to involve parents as much as possible; in phase 1 there is a newsletter and parent assembly. In phase 2, an information leaflet goes home to parents each day based on the theme covered in the drama session and parents are invited in to the school to watch work in progress during the last two drama sessions of the week. In phase 3, parents set goals at home with their child which are written up and sent home along with the HeLP ‘80/20’ fridge magnet. Following this there is another parent assembly. In phase 4, following the 1-1 goal supporting interview, the children’s goals are, once again, sent home in the post.

**Table 1:** Intervention phases, function, BCTs and the component and agent of delivery

**Intervention phase**	**Function**	**Behaviour change techniques**	**Component (Frequency and duration) and agent of delivery**	
**Phase 1**	Establish relationships with schools, children and families	Provide information on behaviour-health link	Whole school assembly (1x20 mins)	HeLP Coordinators
**Creating a supportive context**				
	Raise awareness and increase knowledge	Provide information on health behaviour link	Newsletter article	HeLP Coordinators
			Literacy lesson (to create HeLP rap.poem) (1x1 hour)	Class teacher
Spring term (yr 5)	Promote positive attitudes and norms towards healthy eating and physical activity	Modelling/demonstrating behaviour		
			Activity workshops (2x1.5 hours)	Professional sportsmen/dancers
		Prompt identification as a role model		
Jan-March				
	Increase self-efficacy for behaviour change		Parent assembly (1x1 hour) involving child performances	Class teachers/HeLP Coordinator/Drama group
		Provide information on behaviour-health link		
		Skill building		
**Phase 2**	Strengthen relationships with schools, children and families	Provide information on health behaviour link	Education lessons (5x1 hour) (morning)	Class teacher
**Intensive healthy lifestyles week –** one week				
		Problem solving/barrier identification Modelling/demonstrating behaviour	Drama (5x2 hours) (afternoon)	Drama group
	Increase knowledge			
	Increase self-awareness			
Summer term (yr 5)			(forum theatre; role play; food tasting, discussions, games etc.).	
	Increase self-efficacy			
April-June	Develop communication and problem solving skills	Prompt identification as a role model		
	Increase social support (school, peer and family)	Communication skills training		
		Teach to use prompts and cues		
**Phase 3**	Increase awareness of own behaviour	Self-monitoring	Self-reflection questionnaire (1x40 mins)	HeLP Coordinator/Class teacher
**Personal goal setting with parental support-** goals set during week following drama		Goal setting (behaviour) Problem solving/barrier identification	Goal setting sheet to go home to parents to complete with child (1x10 mins)	HeLP Coordinator/Parents
	Increase self-efficacy for change			

	Develop planning skills	Plan social support		
	Increase parental support	Provide information on where and when to perform a behaviour	1:1 goal setting interview (1x10 mins) (goals sent home to parents)	
Summer term (yr 5)				HeLP Coordinator
June-July		Agree behavioural contract	Forum theatre assembly (1x1 hour)	HeLP Coordinator/Drama group
		Prompt identification as a role model		
**Phase 4**	Increase self-awareness and prioritise healthy goals.	Provide information on health behaviour link	Education lesson (1x1 hour)	Class teacher
**Reinforcement activities**				
	Consolidate social support.			
				Drama group/HeLP Coordinator
			Drama workshop (1x1 hour). Followed by a class delivered assembly about the project to rest of school (1x20 mins).	
		Modelling/demonstrating behaviour		

		Prompt identification as a role model		
Autumn term (yr 6)	Develop monitoring and coping skills			
	Increase parental support			
		Provide social approval		
			1-to-1 goal supporting interview to discuss facilitators/barriers and to plan new coping strategies (1x10 mins).	HeLP Coordinator
		Prompt self-monitoring		
		Prompt intention formation		
Sept-Dec		Follow up prompts		
		Prompt review of behavioural goals	(renewed goals sent home to parents)	
		Prompt barrier identification and resolution		
		Coping plans		

As the Table 1 shows, HeLP is delivered by a combination of personnel. Piloting of the intervention in the early stages identified the actors and the HeLP Coordinator (HC) as key to programme delivery. The role of the HC is to build trusting and supporting relationships, which requires specific competencies, understanding and interpersonal skills such as the ability to listen reflectively and empathise with children, parents and teachers. HeLP should be delivered in a collaborative manner (and has been carefully designed to promote this) to *evoke* rather than *install* motivation with the emphasis on identification with and ownership of the healthy lifestyle messages in order to create the conditions for change. The actors who deliver the interactive drama workshops are highly skilled in the drama techniques used, having completed a week of intensive training prior to delivery of the Healthy Lifestyle Week. All the activities and scripts for this week have been manualised.

The class teacher is specifically tasked with delivering the Personal Social and Health Education (PSHE) lessons prior to the interactive drama during the Healthy Lifestyles Week and a literacy rap lesson during Phase 1, so that they are able to engage with the content of HeLP and use it in other aspects of their teaching if they so choose. All lesson plans link to National Curriculum objectives for PSHE and have been manualised in a booklet with all associated resources on a CD Rom. All worksheets for these lessons are photocopied and prepared in advance by the HC.

In phase 1, professional sport people and dancers are used to talk to the children about the importance of healthy lifestyles and to run practical workshops. This creates a buzz in the school and sets a positive atmosphere for future activities. Children then showcase the skills they learn during these workshops in a parent assembly at the end of phase 1 where they are given further information about the programme by the HC. All components have been manualised so that delivery is standardised, however, HeLP has been designed to allow for some flexibility so that each activity can fit the context of the school, whilst still remaining true to HeLP. For example, schools are able to select the timings of parent assemblies which may occur in the morning or at the end of the school day. The HC works with the school to understand how best to engage and involve the parents which can vary depending upon the type of school.

### The exploratory trial

**Figure 1:**
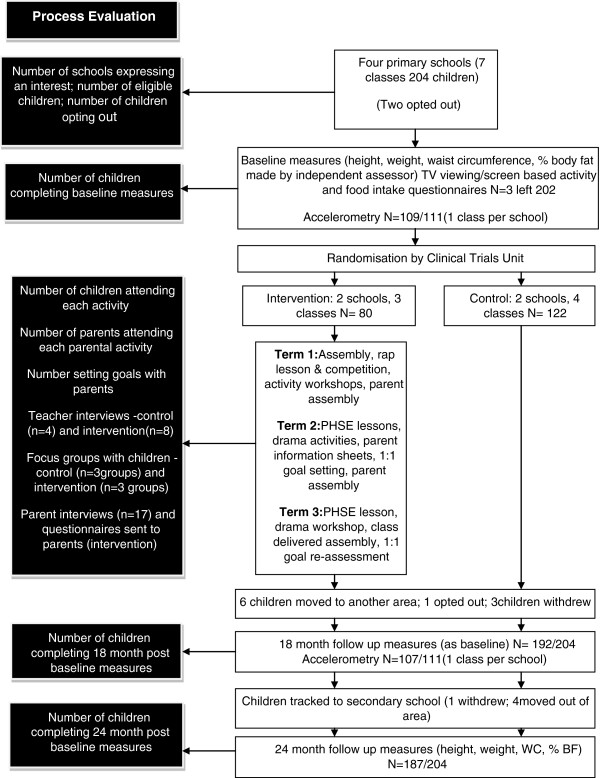
Flow diagram of the exploratory trial.

Pilot 3 was an exploratory randomised controlled trial [32,33] which sought to assess for schools, children and their families; recruitment and retention in control and intervention schools; feasibility and acceptability of HeLP; feasibility and acceptability of future trial outcomes and facilitators and barriers to the uptake of HeLP.The trial took place in Exeter (a city in the South West of England) involving 202 9-10 year old children. There is little ethnic mix in the South West, with the majority of the population being ‘white’. Although overall socio-economic status for the area is higher than average, within Exeter there are some areas with quite severe deprivation. All state Primary and Junior schools in Exeter were eligible to take part if they had at least one single age year 5 class (9-10 year olds) (i.e. not mixed classes, 8-10 or 9-11 year olds). Of the 11 eligible schools in Exeter, eight expressed an interest from which four schools (with a total of 7 Year 5 classes) were randomly selected to participate in the exploratory trial. Following baseline measures, schools were randomly allocated to intervention or control using a telephone based randomisation service involving a statistician independent of the research. All parents of children were sent an information pack with an opportunity to opt out of the study. If the opt out form was not returned within 2 weeks consent was inferred. The class teacher gave daily oral reminders to the children over this 2 week period to ensure that they and their parents had read the information sheet. Figure 1 shows the flow of schools and children through the trial including details of the process evaluation that accompanied the trial.

The behavioural and weight status outcomes of the exploratory trial have already been published [33] so this paper will present:

• Recruitment and uptake in control and intervention schools

• The feasibility and acceptability of the trial design (including the outcome measures)

• Whether HeLP is feasible and acceptable to schools, children and their families

• Whether HeLP is able to engage schools, children and their families.

**Figure 2:**
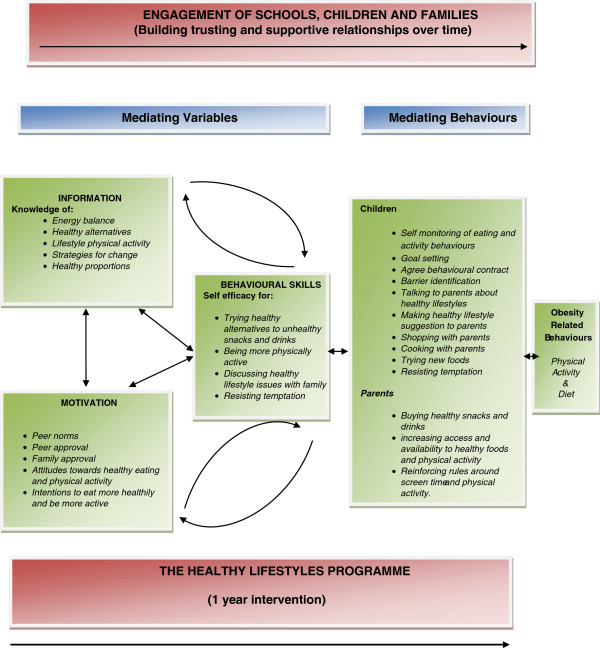
The HeLP process model.

Following the presentation of these results the paper will discuss how these findings informed the development of a HeLP process model (Figure 2) and their implications for the process evaluation for the definitive trial (now running in 32 schools across Devon) [34].

## Methods

The methods utilised in this study conform to qualitative research review guidelines (RATS) guidelines [35].

Ethics approval for all pilots (including the exploratory trial) was granted from the Peninsula College of Medicine and Dentistry Ethics Committee (PCMD).

### Recruitment and uptake

Schools were recruited via the local network of primary school head teachers using a briefing meeting to present the trial and answer any questions. Parents of all children in the year 5 were sent a parent information pack, detailing the trial and what it would mean to their child if the school was randomised to receive HeLP or act a control school. An opt out form was included for those parents who wished to opt their child out of the trial which was returned to their class teacher for collection by the HeLP Coordinator. Participating schools, children and opt outs were recorded on the HeLP database. In intervention schools, uptake of HeLP components (children) and attendance at specific events (parents) was recorded using registers. All information was inputted onto the database.

### Feasibility, acceptability and engagement

#### *i. Children*

For children this was assessed using focus groups consisting of approximately 6 children per group. In the intervention schools, children were sampled by gender and level of engagement with HeLP (based on HC observations) in order to include children with a range of engagement levels. Level of child engagement was judged based on participation in the drama activities during the Healthy Lifestyle Week and parental agreement of goals (determined from parental signature and indication of support).

In control schools, children were sampled by gender only.

The focus group questions for the intervention children related to both the trial design and outcome measures and the HeLP Programme. The control children were only asked questions related to the trial design and outcome measures. The questions can be seen in Additional file 1.

#### *ii. Teachers*

In the intervention schools, all teachers involved in delivering components of the intervention were interviewed including those from the senior management team with whom there had been contact throughout delivery.

In the control schools the year 5 and year 6 teachers involved in the trial were interviewed as both were involved in the study during data collection.

Interview questions in the intervention schools related to both the feasibility and acceptability of the trial design and outcome measures as well as the HeLP programme. The control teachers were only asked questions relating to the trial design and outcome measures. The questions can be seen in Additional file 2.

#### *iii. Parents*

All parents of children who had participated in HeLP were sent a questionnaire. Questions can be seen in Additional file 3. In an attempt to improve the response rate, returned questionnaires were entered into a prize draw for a £25 shopping voucher. Parents were given a stamped addressed envelope to return the questionnaire and a return slip to indicate whether they would be happy to be interviewed. The questions can be viewed in Additional file 4.

All interviews (teacher and parents) and focus groups (children) were audio-recorded. Interviews were carried out by JL and focus groups were carried out by JL and KW.

### Data analysis

Due to the specificity of the questions related to the trial design and outcome measures, the responses of children and teachers were scrutinised for possible barriers to participation. The interview (teachers and parents) and focus group (children) data relating specifically to HeLP (intervention schools only) were analysed using a framework analysis approach [36] as the questions were partially driven by pre-determined concepts. Interview and focus group transcripts from children and teachers in the schools receiving HeLP were read and re-read and an index of multiple emerging themes and subthemes were constructed. Each interview or focus group transcript was coded using the index, and the data represented by each theme were extracted and collated into charts to facilitate the organisation of the data. A summary of each theme was derived from chart entries and direct quotations were identified which represented the range of views expressed in relation to each theme and subtheme. The framework analysis was led by JL with a sample of transcripts analysed independently by KW.

Data relating to HeLP from the children, teachers and parents were triangulated in order build a holistic picture of feasibility, acceptability and engagement.

## Results

### Recruitment and uptake

Two children opted out prior to baseline measures and randomisation. The intervention group consisted of two primary schools (n = 80). School A had 384 children on the school roll with 2.6% eligible for free school meals (FSM) and involved two year 5 classes (n = 59). School B had 170 children on roll (13% FSM) and involved one year 5 class (n = 21). The control group consisted of two primary schools (n = 122). School C had 317 on roll (14% FSM) and involved one year 5 classes (n = 31). School D had 364 on roll (6% FSM) and involved three year 5 classes (n = 91) (See Figure 1).

As all HeLP activities occur during the school day, uptake by the children was high (90-100%). Over 90% of parents in both schools participated in at least one ‘parental engagement’ activity (parent events and goal setting) with 97% (school A) and 66% (school B) of parents agreeing goals with their child.

#### *i. Focus groups with children*

In intervention schools (A and B), three focus groups were carried out with a total of 25 children. In control schools (C and D), three focus groups were carried out with a total of 22 children. Although children were not sampled by weight status, as it was deemed ethically inappropriate to take such an approach, each focus group included children with a range of weights.

#### *ii. Teacher interviews*

In school A, two year 5 and two year 6 teachers and the Assistant Headteacher were interviewed (who was also a parent of a child receiving HeLP). In school B, one year 5, one year 6 teacher as well as the Head were interviewed (n = 8 interviews in intervention schools). In school C the year 5 and year 6 teacher were interviewed and in school D the three year 5 teachers were interviewed together as were the three year 6 teachers (n = 4 interviews in control schools).

#### *iii. Parent Questionnaire and parent interviews (intervention only)*

In total, 40% of parents (32/80) returned the parent questionnaire (24 from school A and 9 from school B) and 60% (19/32) indicated that they were happy to give an interview about the Programme. Seventeen parents were interviewed in total (9 from school A and 8 from school B). All participants were mothers and interviews took place either at the school in a private room or at the parent’s home, depending upon personal preference.

### Is the HeLP trial design (including the outcome measures) feasible and acceptable?

The completion of baseline, 18 and 24 month measures can be seen in Figure 1. Schools were keen to participate and parents were happy for their child to participate even though, at the point of recruitment, group allocation had not been determined. Only two children were opted out prior to baseline measures and 97% and 92% of children were followed up at 18 and 24 months (where children were measured in the first term of their secondary school) respectively. One class per school wore the activity monitor for 7 days and, as with the other measures, follow up was high (96% at 18 months). Compliance with wearing the activity monitors was also good, with useable accelerometry data being obtained from 95% and 85% of participants at baseline and 18 months [33].

Although teachers commented that taking outcome measures during class did take away some curriculum time, they felt that the relationship they and the children had built up with the HeLP Coordinator, who fitted in with their timetable as much as possible, made it easier for everyone. All teachers agreed that the use of email and short meetings at the end of the day to book in measurements dates worked well, and, as they thought the research to be important, were happy to be involved. All teachers felt that they had been given enough information regarding the trial.

Teachers were happy to be interviewed at the end of year 5 and year 6, however, as the relationships in control schools weren’t as strong as those in the intervention schools, intervention teachers were more forthcoming in giving up their time for them. As a result teacher interviews for control schools were carried out in groups if there was more than one year 5 or 6 teacher. Children were happy to participate in focus groups, particularly intervention children who enjoyed talking about the programme. Most teachers preferred these focus groups to occur in a lunch time rather than during a lesson in order to minimise disruption.

In order to present the data for the feasibility and acceptability of HeLP and whether it engaged children, parents and their families, illustrative quotes have been grouped by themes that emerged from the framework analysis, along with relevant evidence from the parent questionnaire.

### Is HeLP feasible and acceptable to schools, children and their families?

Three clear themes emerged which were; the value of HeLP; the compatibility of HeLP with the National Curriculum and HeLP as an enjoyable experience.

#### *i. The value of HeLP*

All eight teachers interviewed believed that HeLP was a worthwhile initiative and that they way in which it was delivered improved understanding and benefitted the children.

*Actually it was good to take the children off curriculum, it really was. Sometimes the intensity of the curriculum overlooks what is relevant and important.* (T1, year 5, female, school A)

*I was very happy for the project to continue. You know it is trying to find a way to get children to understand or an appropriate way for children to understand healthy lifestyles without doing it in a way to dig their heels in.* (T4, Head teacher, male, school B)

All parents who were interviewed indicated that the Programme’s messages fitted with those they were trying to promote at home regarding healthy lifestyles and acted as a prompt to continue promoting these messages.

It is not until your child says something or they are doing something at school and you suddenly think, oh yeah! I have been meaning to do that! It was almost like a prompt to change things.

*I signed up for the Change for Life materials because of it [the project].* (P12, school B)

#### *ii. Compatibility of HeLP with the National Curriculum*

All teachers felt that HeLP was compatible with the National Curriculum and that there were many opportunities to make links to other subject areas and incorporate other activities that promoted HeLP’s messages. None of the teachers commented on an increased workload or that it disrupted the curriculum.

*It* [HeLP] *gave enrichment to the curriculum. It did not impact on our timetable what-so-ever, we were able to accommodate it no problem.* (T1, year 5, female, school A)

*All the time you can keep linking in. I combined the healthy lifestyle messages with the Fair Trade project we were doing. It helps with us being so topic based now as the links are more natural.* (T3, year 5, male, school B)

#### *iii. HeLP as an enjoyable experience*

In the focus groups children were unanimous in their enjoyment of HeLP, particularly the interactive drama activities. This is supported by evidence from teachers and parents who commented on the enhanced self-esteem, sense of control and empowerment they saw in the children which, they felt, was key in creating this ‘feel good factor’. Over a third of parents commented in the questionnaire that they had noticed a positive impact on their child’s self-esteem, mood and/or behaviour as a result of HeLP.

*It was interesting to see the way the children responded to you and the actors. I think it showed a positive side to them that perhaps they weren’t as aware of before because they were labelled as a group who could be problematic. I think that has increased their self-esteem.* (T5, year 6, male, school A)

*What particularly inspired me was how it managed to draw out those children who struggle in some way. The children who shone all had statements of special educational needs. Their behaviour and attitudes around the whole piece of work were outstanding. I think that it was more than just the drama. It is drama with someone who is not your class teacher as those particular children do struggle with behaviour and emotional needs. However good the relationship with the teacher, it is the class teacher who knows about the problems with behaviour and is noticing the negatives. The actors and you did not have that background on the child and the sessions allowed them to over exaggerate and explore whereas in other classroom situations that is not always appropriate.* (T8, Assistant Head, school A)

*T liked it because you took on his feedback and he was listened to about his ideas. He wasn’t being told. He actually said to me that he wasn’t told not to watch telly but it was suggested to watch less. You did not tell him that he couldn’t.* (P15, school A)

### Is HeLP able to engage schools, children and their families?

Engagement with HeLP manifested itself within the following two themes; *‘Knowledge, understanding and awareness’* and *‘taking messages on board’*

#### *i. Knowledge, understanding and awareness of programme activities and messages*

HeLP has been specifically designed, using appropriate behaviour change techniques and creative delivery methods, to encourage children to take the healthy lifestyle messages home to their parents and engage the whole family in discussion. In addition, it aims to ensure that all teachers involved in the delivery of HeLP and at least one member of the senior management team are well informed and supported throughout. HeLP also aims to engage and motivate teachers and the senior management team sufficiently to spread the messages throughout the school in order to build a general awareness.

The data shows that teachers were very aware of the aims of HeLP and the energy balance concept. There was an appreciation of having lesson plans that allowed for adaptability and that delivering these and observing the drama enabled them to see how the messages built up over each phase.

*Every teacher has their own style and they [the PSHE lessons] fitted within their style. I think it was easy to see what the major messages were, so it wasn’t like we were floundering or wondering what you wanted us to do, so that was great.* (T8, Assistant Head, school A)

*As I discussed the project with the children it started to fall into place. I think that we grew in our understanding at the same time as the children did.* (T2, year 5, male, school A)

All the parents who were interviewed were aware of the three key behaviours and the 80/20 message. Most parents were aware of the 4 characters played by the actors, although only half of those parents interviewed could remember which character their child had worked with during the Healthy Lifestyles Week.

*You were trying to promote a healthy lifestyle through diet and exercise and the message came home very strongly about the 80/20 in that you did not have to deny yourself totally but actually, if you made healthy choices 80% of the time, it was probably good enough and also to promote exercise and that certainly did make B aware of how much he was eating and how much exercise he was doing.* (P3, school A)

*I think it was about choices in terms of what they would choose to be doing in their spare time, what they would choose to eat. It wasn’t just about exercise but about making other choices that were healthier and perhaps a little bit more imaginative. I think particularly for [name], he identifies that there were lots of other boys his age that would always choose to go on the computer and use the play station rather than perhaps, you know, apply their imagination to other activities.* (P13, school A)

#### *ii. Taking messages on board*

There was strong evidence from all sources (teachers, parents and children) that both the children and their parents engaged with HeLP. Four sub themes emerged which were; a) initiating discussion with friends and family about healthy lifestyles, b) being more accepting of rules regarding screen time and healthy eating at home, c) being more co-operative and taking greater responsibility and d) the importance of mode and agent of delivery. Teachers were unanimous that year 5 was the key target age as children are gaining independence but are still amenable to the messages. Teachers also felt that the highly inclusive, interactive drama activities which encourage identification with, and ownership of, the messages was crucial in motivating the children to take the messages home to their families.

*He found the activities engaging. I knew what was going on all the time because he would describe to us the drama and the characters. He is not generally chatty about what he does at school so the fact that he did talk told me the project had engaged him.* (P3, school A)

*If I said these are my rules and this is what we should do and it is in your best interest he would say ‘that is not fair and you are just being mean’. But because it was part of a school project and someone else had endorsed it he actually took [the goals] seriously.* (P7, school A)

I have noticed that my children are now more thoughtful when there is free choice (P10 (T8), school A)

*They loved the acting. For me that is the bit that has really stood out. Our children in year 6, they cling to young adults. They really want to socialise with people like that* [the actors]. *It gives them almost an aspiration to be like them*. (T5, year 6, male, school A)

## Discussion

The process data from the exploratory trial demonstrates that the trial design, outcome measures and intervention are feasible and acceptable to schools, children and their families. Throughout the development of HeLP we were mindful to minimise the potential to widen existing health and social inequalities and to ensure that the intervention was feasible and accessible to schools, children and their families from all socioeconomic backgrounds/status. The qualitative data suggests that HeLP is feasible and accessible to differing socioeconomic backgrounds and that SES does not appear to mediate ‘engagement’ at the level of the child. However fewer parents from the less affluent school set goals with their child, although they were able to attend more events than parents from the more affluent school. Moreover the data suggests that HeLP engages those children with lower self-esteem. Indeed a sense of connectedness, good communication and perceptions of adult caring (all of which are fundamental to HeLP) have been shown in studies of schools and families as being related to a wide range of behavioural and mental health outcomes [37]. These findings will be explored further in the definitive trial.

In the main, the responses of participants were extremely positive towards HeLP. The authors believe the reason for this was the invaluable information gained from stakeholders during two years of extensive piloting, involving detailed process evaluations that preceded the exploratory trial. This data enabled improvements and adaptations to be made to the intervention over time to improve the feasibility and acceptability of HeLP for all groups (school, teachers, children and their families) [32]. The authors are aware, however, that parents self-selected for interview and thus may view the intervention in a more positive light than those who did not volunteer. As the interviews were carried out by JL (the HeLP Coordinator) who had built up relationships with the intervention schools in particular, there is the possibility that the teachers and children may have provided socially desirable answers, although they were specifically asked about anything that was difficult or they did not like in relation to HeLP.

Many of the themes and subthemes that emerged from the qualitative data incorporate mediating variables targeted by HeLP and link to the Information, Motivation and Behavioural Skills (IMB) model [28] which was used as a guide in the development of the Programme [26]. These include knowledge, attitudes, self-efficacy, enjoyment, autonomy, self-esteem, social norms, communication and self-monitoring skills.The qualitative data suggest that these variables do not stand alone but interact to strengthen the engagement of children and their parents. We have attempted to capture how we think HeLP engages children and families in the HeLP Process Model (Figure 2). This model attempts to represent the mediating variables related to HeLP within the IMB framework, indicating the feedback loops which appear to strengthen relationships and engagement with HeLP over time in order to create behaviour change. The hypotheses inherent in this process model will be tested as part of the process evaluation for the definitive trial.

The assignment of a HeLP Coordinator for a school was borne out of the understanding that one key contact person with the necessary skills, competencies and training was crucial in building and strengthening relationships with teachers, children and parents over the course of the one year intervention. This led the development team to consider, in depth, the necessary qualities required of the HeLP Coordinator and how best to assess these during recruitment for the definitive trial. In addition, experiential learning has enabled us to produce a detailed training manual for the HeLP Coordinator role, ensuring that delivery is of the quality necessary to engage sufficiently to affect behaviour change.

Relationships were further strengthened during HeLP by using highly interactive drama-based activities within a framework based on four characters with whom the children could identify. Techniques used in the drama sessions allowed children to co-create scenes with the actors and, as such, based learning on the dialogic relationship between fiction and reality, which allows rehearsal for real life [38]. The Confucian aphorism; ‘tell me and I will forget, ‘show me and I may remember’ and ‘involve me and I will understand’ is particularly relevant when choosing delivery methods to engage children with healthy lifestyle messages so that they are motivated to take them home to their parents and discuss them with their peers.

The data show that this method of delivery using well trained actors was shown to be key in engaging the children to; initiate discussion about healthy lifestyles at home, accept family rules around the key messages and increase cooperation at both school and at home. However, despite its potential to empower and engage children, only a few health promotion programmes have primarily or solely involved drama methods in school-based health promotion programmes [39-41]. One reason for this could be that most teachers have only limited experience and therefore lack the ability and confidence to use drama methods [42]. Our data show that quality delivery by those who have the skills is essential and that identification with the four characters played by young actors is the means by which trusting relationships and a bond with the actors can be developed. Stakeholder consultation in the early development phases revealed that primary school teachers do not have the time to attend training sessions in order to deliver such programmes. As HeLP is delivered, in the main, by people external to the school, it does not require teachers to attend training sessions and this was a strong reason for its feasibility and acceptability amongst school staff.

Rather than looking for an active ingredient within HeLP or a set of necessary activities, HeLP aims to affect both upstream and downstream influences on health behaviours hence we wanted the data from interviews and focus groups to help us understand the importance of parental, child and school engagement with HeLP and how best to capture this in the process evaluation for the definitive trial [34].

To enable adaptivity, HeLP has been defined both in terms of what each component is (who is involved and what happens) as well as the intended purpose of each component. Fidelity to both the function and form of HeLP are being assessed in the definitive trial as well as the quality of the delivery which is being assessed on enthusiasm, responsiveness and clarity of messages. The quality of delivery and reach (engagement) for the intervention activities where children, teachers and parents are all together (parent assembly in phase 1 and the forum theatre assembly in phase 3) is being assessed using observations and captured using checklists. The drama workshops in phase 2 (which the data show are key in engaging the children) and the class delivered assembly to the whole school in phase 4 (which can demonstrate the engagement of the participating children as well as the engagement of the children watching) is being assessed in the same way. Engagement is also assessed using records of parental involvement in the setting of goals and by using field notes on the responsiveness of children (during the 1-1 goals setting sessions) and the responsiveness of schools. This will include whether they have incorporated additional activities linked to HeLP’s messages into the curriculum throughout the year long intervention.

Although, not designed as a realist trial [43] it is hoped that by capturing engagement as well as uptake, delivery and mediating variables in the definitive trial, will let us go some way in answering the question ‘what works, for whom and it what circumstances’.

## Conclusions

The qualitative data from the exploratory trial have provided us with evidence of the feasibility and acceptability of HeLP and *how* it engages schools, children and their families. In addition, the data have helped us understand how we can best capture the relationships, processes and activities which is crucial in assessing the programme’s ‘reach’ in the definitive trial, thus helping us to understand the necessary conditions for sustained behaviour change.

## Abbreviations

HeLP: Healthy Lifestyles Programme; FSM: Free school meals; IMB model: Information, Motivation and Behavioural Skills Model; BCT: behaviour change technique; HC: HeLP Coordinator; PSHE: Personal Social and Health Education.

## Competing interests

The authors declare that they have no competing interests.

## Authors’ contributions

JL drafted the manuscript with KW providing critical revision. JL and KW designed the process evaluation and carried out interviews with teachers/parents and focus groups with children. JL analysed the data with KW providing support. JL will act as guarantor of the paper. Both authors read and approved the final manuscript.

## Pre-publication history

The pre-publication history for this paper can be accessed here:

http://www.biomedcentral.com/1471-2458/14/578/prepub

## Supplementary Material

Additional file 1 Focus group schedule. Click here for file

Additional file 2 Teacher interview schedule. Click here for file

Additional file 3 Parent questionnaire. Click here for file

Additional file 4 Parent interview schedule. Click here for file
