# Supplementary material for: Qualitative findings from an exploratory trial of the Healthy Lifestyles Programme (HeLP) and their implications for the process evaluation in the definitive trial
Source: BMC Public Health. 2014 Jun 9;14:578. doi: 10.1186/1471-2458-14-578 (PMC4071326; doi:10.1186/1471-2458-14-578)
Supplement: Additional file 1 — Focus group schedule. [file 1471-2458-14-578-S1.docx]

Focus group schedule for all children:

Questions for control and intervention children:

- At the beginning of the project we sent out an information pack about the study to your parents, you do you remember whether they talked to you about it and did they ask you whether you wanted to participate?
- What do you remember about the project?
  - What did you like most about it?
  - Is there anything thing that you didn’t enjoy so much?
- Do you remember the weighing and measuring lesson?
  - Would you like us to have done that differently?
- Do you remember wearing an accelerometer?
  - Do you remember anything good or not so good about wearing it?
- Do you remember completing the questionnaires?
  - Was there anything that was not so good about completing them?
- Does knowing you will receive a high street voucher have an effect on your participation?
- Do you have any questions?

Questions for intervention children only:

- Did you discuss any part of the project with others at school?
  - Explore what was discussed.
- Did you talk about the project at home with any of your family?
  - Explore what was discussed and when.
- Did you managed to stick to some/all of your goals?
- Was it difficult? Why? Did anyone help/remind you?
- What has been the most helpful part of the project in helping you to make small changes?
- Did you use any special ways of your own to help you stick to your goals? What were they?
- Have there been any changes in your household as a result of the project? If so, what are they?
- Have you noticed any changes in the school as a result of the HeLP?
- What is the main message you will take from this project for the future?
- If you were to give advice to another child of your age to help them lead a healthier lifestyle what would it be?
