# Supplementary material for: Qualitative findings from an exploratory trial of the Healthy Lifestyles Programme (HeLP) and their implications for the process evaluation in the definitive trial
Source: BMC Public Health. 2014 Jun 9;14:578. doi: 10.1186/1471-2458-14-578 (PMC4071326; doi:10.1186/1471-2458-14-578)
Supplement: Additional file 2 — Teacher interview schedule. [file 1471-2458-14-578-S2.docx]

Interview schedule for the teachers:

Questions for teachers from schools allocated to control and intervention

- At the time how did you feel about being approached to be involved in this project?
- Did you feel you were given enough information to understand what the project was about and to make an informed decision about being involved?
- If there were any opt outs, explore why the teacher thinks these children were opted out and how we can minimise this in future work. Explore the possible effect this may have had on these children.
- How did you think the measurement lesson went?
- Did you think there were any negative effects of us weighing and measuring the children?
- Could we have done anything differently?
- How did you think the questionnaire lessons went? Could we have done anything differently?
- Did the wearing of accelerometers affect the children in any way? Did any parents approach you with any concerns about their children wearing them?

Questions for teachers from intervention schools only:

- How did you find hosting the HeLP programme?
  - How did the planning and delivery of it affect your workload?
  - What would have made it easier?
- What did you think of the assembly to introduce the project (giraffes, Q/A with the children, miming the energy balance concept) and the poem/rap competition?
- What did you think of the Exeter Chiefs visit?
- What did you think of the dance workshop?
- What did you think of the parents’ evening? (Introduce rationale, components of project, drama schedule, pupil performances)
- Did these activities increase your workload a lot?
- How did the uptake compare to other parent events? Uptake for this evening (March) was much better than the forum theatre evening (July). Do you have any suggestions why this might have been?
- How did you find the PSHE lessons in the Healthy Lifestyles Week?
- Were you able to watch the drama and if so what did you think of it?
- What did you think of the goal setting?
- What did you think about the parent’s evening in the format of forum theatre?
  - Poor uptake – any reasons why this might have been?
- Did you feel the project had an effect on the whole school?
  - If so, how?
  - If not really how can we reach the whole school more?
- Did you notice any effect of the programme on any of the children/parents?
  - Did it affect boys and girls differently?
- Was it right to include all Year 5 children?
- Is there anything else we could we have done differently?
