# Supplementary material for: Qualitative findings from an exploratory trial of the Healthy Lifestyles Programme (HeLP) and their implications for the process evaluation in the definitive trial
Source: BMC Public Health. 2014 Jun 9;14:578. doi: 10.1186/1471-2458-14-578 (PMC4071326; doi:10.1186/1471-2458-14-578)
Supplement: Additional file 3 — Parent questionnaire. [file 1471-2458-14-578-S3.docx]

**The HeLP Programme**

**Parent Questionnaire**

**INTRODUCTION**

As you are aware, your child has been in a class that volunteered to participate in the Healthy Lifestyles Programme which aimed to deliver healthy lifestyle messages through a number of activities over the spring and summer term. We thank you for allowing your child to participate and hope that it has been a positive experience for them. As your views are extremely important in enabling us to evaluate and develop the programme we would be grateful if you can spare the time to complete this simple questionnaire. There are no right or wrong answers; all we ask of you is to respond to each question as honestly as possible.

Please tick one of the circles to the questions below:

**Were you aware of the following aspects of the Healthy Lifestyles Programme?**

**YES NO**

The 3 key messages 🌕 🌕

The 80/20 message 🌕 🌕

The drama workshops 🌕 🌕

The activity workshops 🌕 🌕

The information sheets 🌕 🌕

**Did you and your child talk about the healthy lifestyles study at all?**

YES 🌕 NO 🌕

**Did you attend any HeLP events?**

YES 🌕 NO 🌕

If so, which events did you attend?

**Were you unable to attend events because:**

They were held at an inconvenient time 🌕

You had other commitments 🌕

They did not appeal 🌕

**Where you happy to set goals with your child using the goal setting sheet?**

YES 🌕 NO 🌕

**If you received the parent information sheets were they useful to you?**

YES 🌕 NO 🌕

**Did they provide any new information for you?**

YES 🌕 NO 🌕

**Did you notice any change in your child’s (please tick if you did)**

Choice of snacks 🌕

Choice of drinks 🌕

Screen time? 🌕

**If you noticed any change could you tell us what it was please?**

**Did you or any other members of your family change your behaviour as a result of the study? If so please can you give details?**

**Thank you very much for completing this questionnaire.**

**Please return it in the stamped addressed envelope provided**
