# Supplementary material for: Qualitative findings from an exploratory trial of the Healthy Lifestyles Programme (HeLP) and their implications for the process evaluation in the definitive trial
Source: BMC Public Health. 2014 Jun 9;14:578. doi: 10.1186/1471-2458-14-578 (PMC4071326; doi:10.1186/1471-2458-14-578)
Supplement: Additional file 4 — Parent interview schedule. [file 1471-2458-14-578-S4.docx]

**Parent Interview Schedule**

- Can you broadly describe the messages you think we were trying to give the children?
- Where possible we tried to invite parents to attend certain events. Were you able to attend any of them and if so what was your impression of them?
- At the end of the HLW with the drama sessions, the children were asked to set 3 goals around the Programmes messages. How did it go? Was the GS sheet (show them) easy to follow? (show parents a copy of the child’s goals from year 5 and 6).
- Do you remember whether your child was able to achieve some of their goals if only for a short time?
  - Probe reasons for this? *Refer to questionnaire*
- Is your child managing to maintain any of their goals? If so, which ones and what do you think keeps them motivated? *Refer to questionnaire*
- Was the HeLP Programme something your child wanted to talk about with you or other members of the family? If so, find out what parts of the project/specific information the child discussed.
- Have these conversations had any impact on the family? If yes, probe further. E.g. Have the family made changes?
  - Have they been receptive to this?
  - Have there been any positive/negative effects as a result?

Are the family/siblings still managing to maintain these changes? If yes, probe why and if no, probe the barriers. *Refer to parent questionnaire.*
